# Surgical Management of Severe Bilateral Breast Silicone Granulomatosis: A Case Report

**DOI:** 10.7759/cureus.85043

**Published:** 2025-05-29

**Authors:** Fanta John, Diana A Fisler, Donovan Valentin Torres, Kimiya Taji, Shay B Dean

**Affiliations:** 1 Plastic and Reconstructive Surgery, St. George's University School of Medicine, True Blue, GRD; 2 Plastic and Reconstructive Surgery, Dean Plastic Surgery Associates Inc., Los Angeles, USA

**Keywords:** autologous breast reconstruction, breast augmentation, granuloma, mastectomy, nipple reconstruction, plastic and reconstructive surgery, silicone granulomatosis, silicone granulomatosis treatment, tissue expander

## Abstract

Silicone granulomatosis is a chronic inflammatory response induced by the introduction of silicone into soft tissue, typically occurring during cosmetic or reconstructive procedures. Despite its well-established association with silicone injections, managing silicone granulomatosis poses significant challenges, necessitating a multidisciplinary approach to achieve favorable patient outcomes. Treatment involves a spectrum of medical modalities alongside surgical interventions, with the extent of surgical management varying based on disease severity. However, recurrence remains a concern, particularly in cases of extensive tissue dissemination following incomplete excision. We present the case of a 46-year-old woman with severe bilateral silicone granulomatosis of the breast treated using a multi-step surgical approach. Our strategy encompassed bilateral mastectomy, bilateral autologous breast reconstruction utilizing the latissimus dorsi pedicle flap technique, tissue expander placement, silicone gel implant insertion, and bilateral nipple reconstruction. This comprehensive approach aimed not only to eradicate granulomatous tissue but also to reconstruct and restore the affected breasts, thereby enhancing both functional and aesthetic outcomes.

## Introduction

Breast augmentation, medically referred to as augmentation mammoplasty, is a surgical procedure designed to enhance breast volume, contour, or symmetry for either aesthetic or reconstructive purposes. It is most commonly performed by board-certified plastic surgeons with specialized training in cosmetic and reconstructive breast surgery. The procedure involves creating an incision at the perimeter of the areola (periareolar incision), in the natural fold beneath the breast (inframammary incision), or in the underarm (axillary incision), followed by the placement of a saline or silicone gel-filled implant and subsequent closure of the incision [1]. In the United States, the U.S. Food and Drug Administration (FDA) regulates breast implants to ensure safety and efficacy, approving a range of devices that vary in size, shape, and surface texture [2].

Driven by factors such as lower cost, easier access, and misinformation about safety, some patients turn to unapproved methods for breast augmentation, notably the direct injection of free silicone. This practice is medically unsafe and widely discouraged due to its high risk of complications. Reported adverse effects include silicone migration, chronic pain, granuloma formation, infection, abscesses, tissue necrosis, and visible disfigurement [3-5]. The immune system may also respond aggressively, leading to conditions such as capsular contracture and silicone granulomatosis, both of which can cause persistent inflammation and significant tissue distortion [6-9].

This case report examines the adverse outcome of bilateral breast silicone granulomatosis in a patient who chose direct free-silicone injection as a method of breast augmentation in Mexico. We provide an in-depth analysis of the clinical progression and characteristics of bilateral breast silicone granulomatosis, while also outlining the intricate surgical strategies, inherent challenges, and long-term management plans employed. The goal was not only to excise the granulomatous tissue but also to successfully reconstruct and restore the morphology of the affected breasts.

## Case presentation

**Figure 1:**
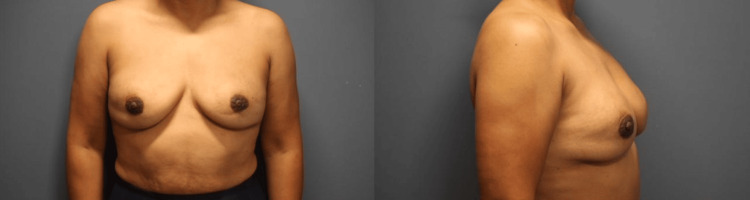
Preoperative photos

A 46-year-old woman presented to our outpatient clinic after she underwent a breast augmentation 10 years ago in Mexico, through the use of direct injections of liquid silicone bilaterally. The patient was no longer satisfied with her augmentation and had been experiencing mental distress as of late. Her breasts had become hardened and caused her diffuse pain. During the physical examination, the patient was noted to have large breasts with multiple firm masses bilaterally, fixed to the anterior chest wall and tender to palpation (Figure 1). There was no warmth or erythema, no nipple retractions or discharge, and the lymph nodes were not palpable. The patient had no chronic health conditions and denied any family history of breast cancer. The patient denied tobacco use and only drank alcohol on occasion.

Before discussing surgical options, the patient underwent imaging studies to further evaluate the extent of breast involvement. An initial mammography showed extremely dense parenchyma in the breasts bilaterally, as well as diffuse, innumerable peripherally calcified oil cysts that are likely attributed to the free silicone injections. This was corroborated by a subsequent bilateral breast MRI with/out contrast, which showed very dense fibroglandular tissue with large focal and confluent areas of increased T1 and low T2 signal as well as mildly enhanced parenchymal tissue bilaterally. No discrete suspicious masses were observed. In addition, there were bilateral enlarged axillary lymph nodes, with the right measuring 1.4 cm in the short axis and the left measuring 1.2 cm in the short axis. Reactive lymphadenopathy was suspected; however, in order to rule out possible malignant lymphadenopathy, an ultrasound-guided core needle biopsy was suggested. The left axillary lymph node was sampled, which predominantly showed small mature lymphocytes and scattered histiocytes, favoring reactive changes due to the free silicone.

Since silicone had been directly injected, a skin and nipple sparing procedure might result in continued symptomatology, given the infiltration of her breast parenchyma with granulomatous tissue. After discussing the risks and potential outcomes of various surgical options, the patient agreed to a two-stage reconstructive approach to minimize complications and recurrence of symptoms. She initially underwent a bilateral total mastectomy with the placement of a tissue expander using acellular dermal matrix and bilateral latissimus dorsi pedicle flaps, followed by the removal of the tissue expander and the placement of silicone implants.

A bilateral mastectomy was performed in order to remove all the granulomatous breast tissue that had resulted from the injected silicone. The excised bilateral breast tissues were sent for histopathological analysis. The standard latissimus dorsi flap raising procedure was followed, keeping the patient in the prone position. The lumbosacral fascia and erector spinae fascia attachments were detached, allowing the muscle flap to be lifted. Dissection proceeded superiorly towards the axilla; perforators were clamped and cut, achieving hemostasis and isolating the pedicle. The donor site was irrigated, and two closed suction drains were placed and brought out at the anterior axillary line before closing the donor site. The left latissimus dorsi flap was done in a similar manner, and both donor sites were covered with bacitracin and xeroform gauze (3% bismuth tribromophenate). The patient was then turned over onto a supine position and redraped. The right breast pocket was irrigated, after which the pectoralis major was lifted and detached inferiorly and medially in order to create a subpectoral pocket. The flap was then dissected from the axilla and brought anteriorly into the breast pocket and inset over a 535 mL MENTOR™ ARTOURA™ Breast Tissue Expander (Mentor Worldwide LLC, Irvine, California, United States) that was sutured to the chest wall. The pectoralis major and latissimus dorsi flap were used to cover the expander and were sutured to the inframammary fold and to each other. The skin pedicle was anchored and sutured into place, at which point another closed suction drain was placed facing the anterior axillary line. Then the left breast was reconstructed similarly. A total of 180 mL of saline mixed with methylene blue was injected into each expander, and the wounds were dressed. There were no procedural complications, and the patient was in stable condition.

Histopathological analysis revealed bilateral benign breast tissue exhibiting a prominent foreign body giant cell reaction indicative of silicone exposure. This reaction was accompanied by chronic inflammatory infiltrates, areas of fat necrosis, and the presence of calcifications. Additionally, approximately 35-40% of the tissue examined displayed grey-white fibrosis, suggesting a significant degree of tissue remodeling in response to the presence of silicone. These findings are consistent with silicone granulomatosis, a condition characterized by the body's reaction to silicone particles introduced into the tissue.

The patient recovered well postoperatively without complaints, and the latismus dorsi pedicle flaps remained viable with their drains appropriately having suctioned serosanguinous fluid. The patient continued wound dressing changes with polysporin ointment and xeroform gauze for two weeks. The two drains were removed one week after the surgery, and the remaining drains were removed at 11 days. We began tissue expansion on the third week, injecting 120 mL of saline into each expander and then again two weeks later, for a total of 420 mL of saline in each tissue expander. At this point, the patient was ready for the next stage of her reconstructive surgery and wanted a revision of the donor site scars that had hypertrophied.

After four months, we removed the patient’s tissue expanders and replaced them with 525 mL silicone MENTOR™ MemoryGel™ Xtra Breast Implants (Mentor Worldwide LLC) in the breast pockets previously created. Additionally, donor site hypertrophic scarring was excised bilaterally (10 cm x 10 cm each), and a complex layered closure was performed, bringing the dermis and subcutaneous tissue together while relieving some tension from the epidermis. Postoperatively, the patient performed wound dressing similar to before, and had her drains removed after two weeks. Given her previous hypertrophic scarring, the patient began using silicone sheets for scar management, and a six-session laser therapy was performed with one session every three weeks for optimal outcome of breast scarring. 

**Figure 2:**
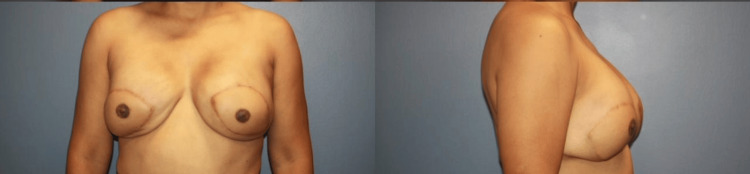
Postoperative photos after reconstruction

The patient’s incisions healed well with good cosmesis after the laser therapy. She noted pain in her right breast near the axillary region with tenderness on palpation. Residual pain or long-term soreness is an expected symptom given the latissimus dorsi flap reconstruction, and the patient was counseled on allowing additional time to recover. Physical therapy was also recommended to improve her strength and range of motion. Four months after her silicone implant placement, the patient had bilateral nipple reconstruction with skate flaps (5 cm x 2 cm each). There were no complications, and sutures were removed after a week. Three weeks later, the patient had areola tattooing for a natural aesthetic appearance. This marked the final step of her bilateral breast reconstruction, and the patient was satisfied with her outcome and had little to no residual pain (Figure 2).

## Discussion

Breast silicone granulomatosis presents a clinical challenge. The condition, characterized by chronic granulomatous inflammation in response to silicone material, can mimic other breast pathologies [10], leading to diagnostic difficulties and necessitating a multistep approach for effective management. Similar cases have been reported in the literature, highlighting the variable presentation and challenges associated with both diagnosis and treatment [11].

In prior studies, patients with silicone granulomatosis have been managed using a combination of medical and surgical interventions [12]. Medical approaches, including intralesional corticosteroid injections, systemic steroids, and immunomodulators, have been employed to modulate the immune response and reduce inflammatory reactions associated with silicone leakage [13,14]. However, these methods often provide only partial relief and are typically adjunctive to surgical intervention [12,13]. Surgical excision remains the primary modality in managing advanced cases, though it presents significant challenges due to the widespread dissemination of silicone within tissue planes [12,13]. Other case reports have also described incomplete excision as a common limitation, with residual silicone potentially leading to persistent inflammation, recurrent granulomas, and further breast deformity [15,16].

Given these challenges, various reconstructive techniques have been explored to optimize aesthetic and functional outcomes post excision [16-18]. Reports in the literature have documented the successful use of autologous tissue flaps, including the latissimus dorsi flap, deep inferior epigastric perforator (DIEP) flap, and transverse rectus abdominis myocutaneous (TRAM) flap, to address significant volume loss and contour irregularities [17,18], such as observed after extensive excision of granulomatous tissue, with best outcomes if breast reconstruction is performed immediately after mastectomy [15].

The publication of case reports enhances the understanding of conditions like breast silicone granulomatosis. Each documented case expands the collective knowledge base, helping clinicians improve treatment approaches and patient outcomes. As more cases are reported, patterns in effective management may emerge, supporting the development of standardized treatment guidelines.

## Conclusions

This report details the complex, multi-phase surgical intervention required to manage severe bilateral silicone granulomatosis resulting from direct free-silicone injections during breast augmentation. While direct free-silicone injections may appeal to patients due to their lower cost, accessibility, and misconceptions regarding their safety, the associated risks are profound. In light of these risks, patients in the United States should opt for FDA-approved breast augmentation procedures performed by board-certified plastic surgeons to ensure the use of safe, evidence-based methods that are customized to their specific needs.
